# On Torsion of the Spermatic Cord, with the Report of a Recent Case

**Published:** 1904-12

**Authors:** J. Lacy Firth

**Affiliations:** Surgeon to the Bristol General Hospital


### ON TORSION OF THE SPERMATIC CORD, WITH THE REPORT OF A RECENT CASE.

BY

J. Lacy Firth, M.S.Lond., F.R.C.S.,

*Surgeon to the Bristol General Hospital.*

Torsion of the spermatic cord is a rare condition, and one which has seldom been recognised before the affected parts have been laid bare by operation. I believe most surgeons would consider the possible existence of the condition if confronted with a case in which severe pain and swelling in the groin had attacked a male possessing an imperfectly descended testicle on the affected side. But I suspect that few of us, when considering the diagnosis of acute painful enlargements of the fully-descended testicle, are in the habit of recollecting that the cause of the swelling may be torsion. I confess that in the past such has not been my custom, and that, partly for this reason, when at last I met with a case of testicular swelling from that cause, I failed to recognise its nature. The nearest approach I made to a correct diagnosis, if I may speak of approaching that which I left so distant, was to recognise that the case was peculiar and one which I did not fully understand. Later, finding this mental position uncomfortable, I took the downward step of attributing the swelling to a cause entirely different from the real one. The following are the notes of the case:—

W. S., aged 23, a labourer, came to my out-patient department at the Bristol General Hospital on Friday, August 5th, 1904, complaining of pain and swelling in the right testicle and scrotum. These troubles had begun five days previously without known cause. No strain or blow had preceded their onset. There had been an attack of vomiting at the beginning, but none later. The first symptom had been pain, chiefly in the groin, and the swelling of the testicle had been first noticed a few hours later. For five days the swelling had gradually increased and the pain continued.

Examination showed the right testicle swollen to the size of a large hen's egg and very tender. The organ felt heavy and firm. It was most firm posteriorly. In front, in the upper two-thirds, deep fluctuation could be elicited. The spermatic cord and vas deferens felt absolutely normal above the swelling. The scrotum on the same side was reddened and slightly œdematous.

There was no history of venereal disease, and the urine was clear and free from gonorrhœal shreds or other deposit. Rectal examination showed that the prostate was normal, and that there was no enlargement of the vesiculæ seminales.

After questioning him, the man stated that on two occasions, one two years, the other eight weeks previously, a swelling had descended from the groin of the same side (right), with uneasiness rather than pain, and had remained for a few hours. On each occasion this had been dispelled by rubbing the groin. He described the present swelling as having descended from the groin in a similar way. [I surmise that at least in the present instance the pain in the groin had led to the patient's misconception on this point. During the whole time he was under my observation there was no sign of any abnormality in the groin. It seems not unlikely that the groin troubles on the other two occasions were the result of a slight degree of torsion of the spermatic cord, and that coincidently with the friction the torsion had disappeared.]

The man was first treated as an out-patient, but at the end of ten days, no improvement being manifest, I admitted him as an in-patient. On the eighteenth day of his illness I explored the swelling, which had changed very little, though if anything it had increased a little in size and become slightly redder and more painful, with a needle and syringe, using full antiseptic precautions. The needle was passed twice into the deeply fluctuating area in front. About one drachm of blood-stained fluid was withdrawn. I considered the fluid to be inflammatory effusion in the tunica vaginalis secondary to some form of testicular inflammation. A further period of rest in bed was ordered. During the following ten days discomfort rather than pain was felt in the testicle. On the thirty-second day of the illness ether was given, and I exposed the testicle by incision. For a day or two previously there had been a return of pain, and the skin had become more reddened and adherent to the parts beneath and fluctuation more distinct. At this period I held the view that most probably the trouble was of tubercular origin, the more because there was a strong family history of phthisis.

At the operation the skin and sub-cutaneous coverings of the testicle were found to be œdematous and about three-quarters of an inch thick. The parietal layer of the tunica vaginalis was also thickened, and in some places directly adherent to the visceral layer, in others separated from it by blood-clot. From the uppermost part of the sac, however, about a drachm of turbid yellow fluid escaped, and this had an offensive odour. The testicle and epididymis were soft and mottled greyish-yellow and brownish in colour. Immediately above the testis, in the highest part of the tunica vaginalis, the spermatic cord was found to be twisted, the condition being recognised by the spiral course of the dilated veins and by spiral grooves. It was not difficult to untwist the cord. To completely do this the testis had to be rotated certainly through two half-turns. At the time I estimated the degree of twisting to be three half-turns. The epididymis was a little swollen, but had not a hemorrhagic appearance. It lay obliquely along the uppermost border of the testis, the latter having its long axis almost horizontal. Though the testicle and epididymis had their long axes almost horizontal, there was a slight inclination of these downwards, outwards and backwards, and the globus major was slightly lower than the tail of the epididymis. The twisted part of the spermatic cord, which was entirely within the cavity of the tunica vaginalis, had a vertical length of about half an inch. The testicle, at this stage, the adhesions to the tunica vaginalis having been separated, hung suspended from the top of the tunica vaginalis by a short length of twisted spermatic cord. The twisted cord proved, in short, a pedicle by which the testicle was suspended in the vaginal cavity of the tunic, and the organ had no other attachments. As the testicle was obviously gangrenous, it was removed with its tunic. The spermatic cord was ligatured about half an inch above the twisted portion, where it appeared quite healthy. The wound was closed, except where a small drainage-tube was inserted. Primary union followed, the wound running an apparently aseptic course.

Examination of the parts removed, after preservation for some weeks in formaline solution, shows that the twist was from within out, or in the direction opposite to that of the movement of the hands of a watch. When the cord is untwisted by one half-turn, the globus major and the upper pole of the testicle to which it is attached are brought from an inferior, posterior and external position to one superior, anterior and internal. Another half-turn in the same direction carries the globus major again to a posterior and external position, but leaves the long axis of the testicle and epididymis horizontal. It seems a little uncertain whether the testis was twisted another half-turn during life or not. If the testicle is given another half-twist, *i.e.* a third, in the direction opposite to that of the original twist, the free border of the upper pole of the testicle will be directed forwards; in other words, the testicle will then be horizontal, with the tail of the epididymis directed backwards, that is, will be in a position of combined "anterior inversion" and "horizontal inversion." It is certain that the testicle before torsion of the cord occurred had the abnormal position of "horizontal inversion." I believe now, as a result of a careful examination of the specimen, that the degree of torsion was two half-turns, and that before torsion the testicle had the abnormal position of horizontal inversion, but not that of anterior inversion. In addition, the testicle is in shape more flattened from side to side than normal.

The organs were not cut into until they had been hardened in formaline for several weeks. The section shows that for one-third of an inch at the periphery the testicular tissue is white in colour, but the central parts, the rete testis, and the whole of the epididymis are of a homogeneous, dull brick-red colour. The red colour must be due either to the previously-congested state of the affected parts, or to congestion together with extravasation of blood.

The chief facts of interest in the case recorded above may be summarised as follows:—A young adult, with the testis (right) completely descended, but possessing the anatomical peculiarities of being suspended freely in a horizontal position from the top of the tunica vaginalis by a pedicle of mesorchium, was attacked rather suddenly with severe pain in this testicle, followed in a few hours by swelling of the organ and œdema and redness of the scrotum, and on the thirty-second day, an exploratory puncture having been made on the eighteenth day, the cause of the trouble was found by operation to be torsion of the spermatic cord and gangrene of the testicle.

The first recorded case of torsion of the spermatic cord appears to be that of Delasiauve in 1840. The next, in order of publication, were those of Scarenzio in 1859, Langlet in 1871, Langton in 1881, and Nicoladoni in 1885. Nicoladoni thought he had discovered an undescribed affection when he published his first case, and must have been unaware of the earlier publications mentioned above. To the present time between forty and fifty cases of torsion have been recorded. The best account of the affection with which I am acquainted is that of André Lapointe in a small monograph entitled, *La Torsion du Cordon spermatique et l'Infarctus hémorrhagique du testicule*, 1904, and I must here express my indebtedness to that writer for many of the facts, considerations and references given below.

Torsion of the spermatic cord has been met with in two chief forms, viz., first, that in which the testicle, together with its tunica vaginalis, is rotated, the cord being twisted outside the tunica vaginalis; and, secondly, that in which the cord and testicle are twisted within the tunic. The first form, named *bistournage accidentel* by Lapointe, is comparable to the operative bistournage carried out in certain animals. The second form has been called *volvulus of the testicle* by the same writer. Three cases only of accidental bistournage seem as yet to have been recorded, viz. one each by Delasiauve,[1] Legueu,[2] and Barozzi.[3] In but one of these was the testicle fully descended, and it is possible in that case, and certain in the other two, that the torsion was produced by the sudden and forcible expulsion of an imperfectly-descended testis through the external abdominal ring. Bistournage, as practised on certain animals, consists of two acts. By the first manipulations the adhesions between the scrotal tissues and the external surface of the tunica vaginalis are broken down; by the second

[1] *Rev. méd. franç. et étrang.*, 1840, i. 363.

[2] *Bull. et Mém. Soc. de Chir. de Par.*, July 15th, 1896.

[3] *Bull. Soc. Anat. de Par.*, 1898, lxxiii. 188.

the testicle, together with its tunic, is rotated through at least four complete turns. This degree of torsion obliterates both the veins and arteries of the cord completely, and the testicle passes into a state of anæmic necrobiosis, followed by atrophy. In torsion of the cord as met with in man, on the other hand, the testicle passes into a state of hemorrhagic infarct and subsequently atrophies if the parts remain aseptic, or becomes gangrenous if the parts become septic. The difference in the results of operative bistournage in animals and torsion of the cord in man is due to the difference in the degree of interference with the permeability of the vessels of the cord. In the former the arteries and veins are completely obliterated; in the latter the arterial obstruction is partial and the venous complete, or nearly complete.

Of volvulus of the testis between forty and fifty cases have been recorded. In addition a number of cases have been recorded in which the testicle had passed into a state of hemorrhagic infarction, like that produced by torsion of the cord, but in which no such torsion was found.

*Causation of Volvulus of the Testicle.*—The testicle, with normal anatomical arrangements, rests upon the posterior wall of the scrotum much in the same way as certain parts of the colon rest upon the posterior abdominal wall, and has only a partial covering of serous membrane. There is no mesorchium or mesotestis composed of two layers of serous membrane, and comparable to the mesentery of the small intestine. With these arrangements volvulus of the testicle is probably impossible. The one constant abnormality found in cases of volvulus appears to be the presence of a more or less elongated mesorchium or mesotestis, and the suspension of the testis in the cavity of the tunica vaginalis by this mesorchium. It is this mesorchium, forming a suspensory pedicle for the testicle and containing the spermatic cord, which becomes twisted. This relation of the testicle to its fibro-serous tunic is analogous to that of the heart to the pericardial sac. In one exceptional case, recorded by Lexer[1], the twisted pedicle suspended the testicle but not the epididymis. The torsion had taken place

[1] *Arch. f. klin. Chir.*, 1894, xlviii. 201.

between the globus major and the superior pole of the testicle. The rest of the epididymis was unattached to the testicle, and lay outside the tunica vaginalis.

It is important to remember that the testicle with a twisted cord has been found perfectly descended and imperfectly descended about an equal number of times. The essential cause of torsion is not therefore imperfect descent of the testicle, but, as we have seen above, the formation of a mesorchial pedicle. In its abdominal position, before descent, the testicle possesses a mesotestis or mesorchium. During the normal descent of the organ this mesotestis disappears. With imperfect or delayed descent, just as with complete descent, the mesotestis may or may not persist. The persistence of a mesotestis does not necessarily cause either imperfect or delayed descent of the testicle, though no doubt it favours these occurrences by interfering with the natural action of some of the forces which normally produce descent. Hence a testicle retained in the groin is more likely to have a mesotestis than a testicle which has passed into the scrotum. Many of the victims of torsion of the cord gave no history whatever of imperfection or delay in the descent of the affected testicle. In fact, a testicle provided with a mesotestis may reach the fundus of the scrotum before birth, and therefore without recognisable delay in descent. Possibly a fully-descended testicle with a mesotestis is more liable to torsion than a testicle with a mesotestis remaining in the groin, for in the former case the mesotestis is more likely to be elongated and attenuated.

In addition to having a pedicle, the testicle, in cases of torsion, has frequently had the abnormal position in the scrotum of either "horizontal inversion," or "vertical inversion," or "anteversion." In horizontal inversion the long axis of the testicle and epididymis is horizontal. In vertical inversion the change of position is the same in kind but greater in degree, and the globus major lies below and the globus minor above. In anteversion the epididymis is in front, and the free border of the testis faces backwards.[1]

[1] I have adopted the English and French phraseology for these abnormal positions of the testicle. German surgeons use similar terms in a rather different sense, and by *inversio verticalis* mean that the change of position is

In these cases it would appear that during descent the testis has been more fixed at its lower than at its upper pole. The upper pole has therefore descended relatively more than the lower, and the testicle has passed into the position of horizontal inversion, and either remained in that position or passed from it to that of vertical inversion, pivoting as it were in these movements upon its lower pole. Kocher has shown that when the testicle pivots in this way on its lower pole the descending upper pole drags with it most of the vessels of the spermatic cord, and separates, to a certain degree, a vascular fasciculus from a deferential fasciculus in the cord. With these fasciculi separated in horizontal inversion, producing the so-called "bifurcation of the cord," it is easy to realise that a smaller degree of torsion will effect a greater degree of vascular obstruction, through one fasciculus compressing the other, than if the torsion takes place with the fasciculi close together, as in the normal cord. In some of the recorded cases of torsion with infarction of the testicle the degree of torsion was only one half-turn. Horizontal inversion existed in most of these cases, and affords an explanation of the great effect of the small twist. Various abnormalities of the tunica vaginalis have been found with torsion of the testicle, such as persistence of the funicular process, with or without a hernia. It is obvious that the more roomy the tunica vaginalis the more easily can the testicle within become rotated. Lastly, the shape of the testicle has been abnormal, *e.g.* flatter than usual, or pear-shaped in some cases.

The exciting causes of torsion of the cord are less understood than the disposing causes discussed above. In some cases torsion has immediately followed the strain of lifting a weight, sneezing, blowing wind instruments, falling, jumping, or at stool. A feature common to all these strains is the sudden and forcible contraction of the abdominal muscles. The testicle retained in the groin is more likely to be affected by them than the fully-descended testicle. In many of the cases of torsion no round the vertical axis of the testis, and by *inversio horizontalis* that it is round the horizontal axis, both being either complete or incomplete. Thus the "inversio verticalis" of the Germans is the anteversion of the English writers, and the "vertical inversion" of the English is the complete "inversio horizontalis" of the Germans.—*Vide Lehrbuch der Urologie*, Von Leopold Casper, 1903, p. 323.

history of strain was obtainable. The torsion seemed to occur spontaneously, and in some even during sleep. Possibly contraction of the cremaster muscle has been the cause in such cases, or sudden changes in the state of distension of the veins of the cord (Spencer).[1] It is difficult to see how the gubernaculum could bring about sudden torsion of the cord.

The degree of torsion has varied from a half-turn to three complete turns, and has been about as often in one direction as in the other. Sometimes detorsion has been easily effected or has occurred almost spontaneously, especially when the torsion was recent; in others, when adhesions had formed, detorsion has been difficult or impossible.

The appearances of the affected parts have usually been those of hemorrhagic infarction of both the testicle and epididymis, especially of the latter, the organs being swollen, purple in colour, and showing much extravasation of blood on section. The pedicle has been swollen, turgescent and purple, with distension and thrombosis of the veins. In my case the hemorrhagic state of the testicle was much less striking than in most of the others. There has usually been effusion in the tunica vaginalis, often hemorrhagic. In one case, that of Keen,[2] which was associated with a reducible hernia, pyogenic cocci were found in the fluid around the testicle. The state of the parts described is the result of partial or complete venous obstruction associated with the retention of complete or partial arterial permeability.

*Symptoms.*—The age of patients with this affection has varied from four months (Dowden) to sixty-two years (Nicoladoni). The symptoms comprise those produced locally and those at a distance, the latter being the reflex effects of compression of the nerves in the cord. At first the pain may be lumbar or iliac, but soon passes into the testicle. The abdomen may be rigid and retracted, and the thigh flexed. Vomiting is frequent, and has been known to become stercoraceous (Whipple and Nash).[3] Syncope, pallor and sweating, with a rapid and feeble pulse, are common symptoms. In nearly all cases the onset of severe

[1] *Tr. Path. Soc. Lond.*, 1892, xliii. 51.

[2] *Med.-Chir. Tr.*, 1892, lxxv. 253. [3] *Brit. M. J.*, 1891, i. 724.

symptoms has been sudden, an important diagnostic point. In a few cases, however, the onset symptoms have not been severe, and the patients have then been able to walk and even work for a day or two. Later, in these cases, the symptoms became more severe, and compelled the patients to take to their beds. In another group of cases the symptoms have quickly subsided, but the patients have remained liable to recurrences. In Van der Poel's case, for example,[1] the patient, a medical man, 25 years of age, had had many such attacks, usually during the day, but occasionally coming on at night during sleep. Sometimes he had two attacks in one day, sometimes months passed without an attack. The patient, after a time, discovered that the cause of his trouble was torsion of the testicle, and that he could relieve himself by rotating his testicle in the opposite direction. Gifford Nash[2] has recorded a case in which he successfully relieved a youth with torsion by untwisting the cord. The symptoms had lasted an hour and a quarter. A tender swelling of the cord itself above the swollen testis helped Nash in making the diagnosis. Subsequently the affected testicle became smaller than the other.

*Diagnosis.*—Hitherto a correct diagnosis of torsion of the cord has been rarely made. The confusion has most often been with incarcerated or strangled hernia. But in torsion intestinal obstruction is not absolute, and vomiting not as a rule persistent. It is in the cases of torsion of the imperfectly-descended testicle that confusion with hernia is most likely to occur. The mistake could hardly be made if the testicle were fully descended, unless an irreducible hernia was present at the same time, for otherwise the inguinal canal would be free. If a strangulated hernia of the partial variety (Richter's) occurred in association with an undescended testicle, the difficulties in diagnosis would be very great. Dowden[3] points out that in strangulated hernia the swelling and pain begin almost simultaneously, whereas in torsion the swelling follows the pain.

When the cord of an undescended testicle is twisted the condition has also to be distinguished from the other disorders

[1] *Med. Rec.*, 1895, xlvii. 737. [2] *Brit. M. J.*, 1893, i. 742.
[3] *Scottish M. & S. J.*, 1901, ix. 229.

which may affect such a testicle, viz. from the so-called strangulation of the undescended testis, and from inguinal orchitis. The first of these, strangulation of the undescended testicle, produced, it has been supposed, by the pressure of the pillars of the external abdominal ring upon the testicle suddenly extruded between them, or upon the cord of a testicle so extruded, cannot be distinguished clinically from torsion. Godlee and de Quervain[1] have reported cases of strangulation of this kind without torsion. The condition seems to be an extremely rare one, and it is highly probable that in most of the cases in which the diagnosis of strangulation has been made the real condition present, though unrecognised, was torsion of the cord. It is equally probable that many cases which have been regarded as examples of inguinal orchitis have been really examples of torsion, though naturally a testicle in the groin is not exempt from the forms of inflammation which attack the fully-descended organ, *e.g.* gonorrhœal or traumatic orchitis.

When the cord of a fully-descended testicle has become twisted the condition has to be distinguished from epididymitis, epididymo-orchitis, or orchitis of urethral or other origin, suppurative inflammation of the tunica vaginalis, tubercular disease of the testicle and ruptured varicocele, and acute hemorrhagic infarction of the testicle. The difficulty in these scrotal cases in the past has been no doubt chiefly due to the fact that torsion has been regarded as an affection of the imperfectly-descended testicle only. The local symptoms are not pathognomonic, but the sudden onset of the pain, the rapid swelling of the testicle and its coverings, and the associated abdominal symptoms and collapse should enable one to recognise the disease if its possible existence is borne in mind.

The condition of acute hemorrhagic infarction of the testicle, however, resembles torsion very closely in its clinical course, and could hardly be distinguished. The affection was first described by Volkmann.[2] It seems not unlikely that the case Volkmann described was really one of torsion. But there are four other cases, recorded respectively by Niemann,[3]

[1] Quoted by Lapointe, *Op. cit.*, p. 103.

[2] *Berl. klin. Wchnschr.*, 1877, xiv. 769. [3] *Breslau. aertl. Ztschr.*, 1884, vi. 13.

Englisch,[1] Mayer,[2] and Mauclaire,[3] which show that hemorrhagic infarction of the testicle may occur without either torsion or strangulation at the external inguinal ring. In all these cases the affected testicle was fully descended. The initial symptoms were less abrupt and severe than those of torsion, and this fact might be a help in diagnosis.

*Prognosis.*—No case of torsion of the spermatic cord yet recorded has ended fatally. The testicle itself has, in at least three cases, been saved by timely detorsion. Two cases of this kind have been mentioned above, and a third is recorded by Perry.[4] In all the other cases the testicle has either been removed by castration, or has sloughed, or has atrophied. As pointed out by Lapointe, there are eleven cases on record in which the cord was untwisted by open operation and the testicle not removed. In one additional case the testicle was left to its fate without untwisting the cord by Langton.[5] In that case, and in six of those in which the cord was untwisted, the testicle was subsequently eliminated in a septic state. The records of three only of the remaining five report the ultimate fate of the testicle. The fate was atrophy. They are the cases of Von Meyer,[6] Bryant,[7] and Defontaine.[8] The question arises, Why did the testicles slough in the other six cases? It is significant that in all there had been an operation. No testicle with torsion of the cord has been spontaneously eliminated by septic processes without a previous operation. The fate of a testicle which has undergone hemorrhagic infarction from torsion would seem then to be simple atrophy, partial or complete. If gangrene ensues it is the result of superadded infection. The path of infection may be either the wound inflicted by the surgeon or possibly the blood stream, the contamination of the blood having taken place elsewhere in the body.

1 *Wien. klin. Wchnschr.*, 1893, vi. 603. 2 *Ann. Surg.*, 1897, xxvi. 666.
3 *Ann. d. Mal. d. Org. génito-urin.*, 1902, p. 818.
4 *Birmingh. M. Rev.*, 1898, xliii. 279.
5 *St. Barth. Hosp. Rep.*, 1881, xvii. 188.
6 *Deutsche med. Wchnschr.*, 1891, xvii 800
7 *Med.-Chir. Tr.*, 1892, lxxv. 247. 8 *Arch. prov. de Chir.*, 1894, iii. 141.

*Treatment.*—The methods of treatment available for torsion are as follows:—

(1) Detorsion by taxis.
(2) Detorsion through an incision.
(3) Detorsion, with fixation of the testis in the scrotum (orchidopexy), including transplantation if the testis is imperfectly descended.
(4) Castration, immediate or secondary.

Detorsion without fixation of the testicle is not ideal treatment, for it leaves the patient liable to a recurrence of the trouble. Detorsion with fixation of the testicle in the scrotum in such a way that recurrence will be rendered impossible is the method of choice if it can be satisfactorily carried out. Immediate castration is required, first, if the testicle has become infected and is gangrenous; and, secondly, if detorsion and orchidopexy are found impracticable or have been tried and failed. If a non-infected hemorrhagic testicle is left after detorsion and the wound remains aseptic, a portion of the gland at least may survive to be of use in the economy. If, however, the wound becomes septic, gangrene of the testicle will almost certainly follow and secondary castration become necessary.

